# Secondary analysis of *Staphylococcus aureus* whole genomes reveals diverse antimicrobial resistance profiles

**DOI:** 10.17912/micropub.biology.000903

**Published:** 2024-04-28

**Authors:** Alyssa A Nitz, Daniel L Johnson, Pungki Lupiyaningdyah, Mckay A Meinzer, Joshua S Ramsey, Colin M Robinson, C Sebastian Valencia Amores, Brett E Pickett

**Affiliations:** 1 Brigham Young University, Provo, Utah, United States

## Abstract

Antimicrobial resistance (AMR) in microorganisms is an ongoing threat to human health across the globe. To better characterize the AMR profiles of six strains of *Staphylococcus aureus* , we performed a secondary analysis that consisted of the following steps: 1) download fastq files from the Sequence Read Archive, 2) perform a *de novo* genome assembly from the sequencing reads, 3) annotate the assembled contigs, 4) predict the presence of antimicrobial resistance genes. We predicted the presence of 75 unique genes that conferred resistance against 22 unique antimicrobial compounds.

**Figure 1. AMR gene and resistance profile:**
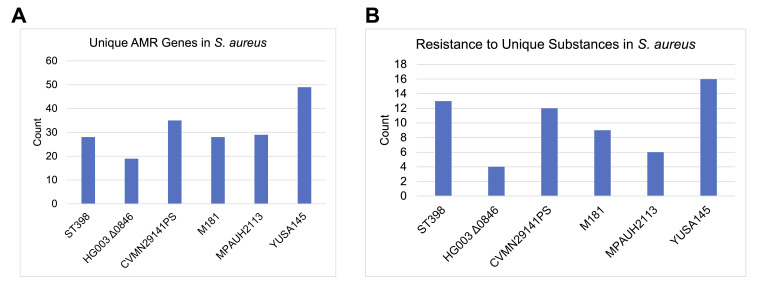
Summarized information for antimicrobial resistance in *Staphylococcus aureus* , including A) the number unique AMR genes for each of the six strains and B) the number of unique substances against which each strain is resistant.

## Description

Antimicrobial resistance is an ongoing threat to global human health ^1–3^ , with increasing prevalence in clinical settings ^4–8^ . Bacteria can gain resistance to a given antibiotic by several known mechanisms including efflux pumps, antibiotic modification/inactivation, modifying the drug target, or bypassing the targeted pathway ^9–11^ . Methicillin-resistant *Staphylococcus aureus* (MRSA), which comprises many nosocomial infections, are being identified and reported more frequently ^12–14^ . As such, the aim of this work was to apply existing tools to better characterize the diversity of antimicrobial resistance genes among six strains of *Staphylococcus aureus* .

We began by downloading the publicly available fastq sequencing files for six *S. Aureus* genomes from the Sequence Read Archive (SRA) at NCBI prior to performing a *de novo* assembly of each genome. We then wanted to predict which antimicrobial resistance (AMR) genes were present in the assembled contigs from our selected genomes. We observed vast differences in the number of AMR genes, and the number of substances to which the bacteria were resistant (Extended Data).

We next examined the diversity of AMR genes in the assembled contigs from these isolates. We calculated the mean number of resistance genes in these isolates to be 31.3 (standard deviation 10.05), and a median number of 28.5. Interestingly, we found that all six strains contained signatures for eight AMR genes including *tet(38)* , *hld* , *aur* , *icaC* , *hlgB* , *hlgC* , *hlgA* , and *mepA* . In this case, we detected genetic signatures for 49 AMR genes in the YUSA145 genome, the highest of the dataset; and a minimum of 19 resistance genes in the HG003 Δ0846 strain.

We then reviewed the number of unique substances that these isolates were predicted to have resistance against. The mean number of unique substances to which these isolates were resistant was 10 (standard deviation 4.52), and a median number of 10.5. As expected, we observed that all six strains were predicted to be resistant to tetracycline; while five of the strains were resistant to beta-lactams and four were resistant to methicillin. We found that the YUSA145 isolate had predicted resistance to 16 unique substances, the highest number in the analysis. This supports prior work that shows the YUSA145 isolate to have strong antimicrobial resistance, including methicillin ^15^ . In contrast, the HG003 Δ0846 strain had resistance to only 4 unique substances.

## Methods

All of the bioinformatics software and tools were installed within modular Conda environments on a Linux cluster ^16^ , an approach that minimized software version incompatibility.

*Data Acquisition and Quality Control Analysis*

Six sets of Illumina paired-end fastq DNA sequencing files of *Staphylococcus aureus* genomes were programmatically retrieved from the Sequence Read Archive (SRA) database, hosted at the National Center for Biotechnology Information (NCBI) ^17^ . This was done using the prefetch and fasterq-dump functions in the sratools software package (version 3.0.10 on Linux) with default parameters. The paired-end Illumina read files were trimmed using TrimGalore! (version 0.6.6 with Cutadapt version 1.18 on Linux) using default parameters other than a Phred score cutoff of 20 and ASCII+33 quality encoding. A panel of quality control metrics were then calculated from the raw reads using FastQC (version 0.11.9 on Linux) using default parameters. Once the quality control analyses for the raw reads were completed and evaluated, a *de novo* assembly was constructed for each set of paired-end reads using SPAdes (version 3.15.5 with Python 3.12.1 on Linux) using default parameters and the --isolate flag ^18^ . The assembled contigs for each isolate are publicly available ( https://doi.org/10.17605/OSF.IO/7D3FH ), with the metadata also available on the project page and as extended data for this study.

*Antimicrobial Resistance Gene Detection*

The AMRFinder tool was used to identify regions in the assembled contigs that contained known antimicrobial resistance (AMR) genes ^19,20^ . This program was run with the following parameters: --plus, -O Staphylococcus_aureus to include genes that have been detected specifically within this bacterial taxon.

## Reagents

**Table:** 

**SRA ID**	**Isolate Name**	**Host of Isolation**
SRR21285297	M181_2017	Homo sapiens
SRR21098652	MPAUH2113	Homo sapiens
SRR3194952	CVM N29141PS	Turkey
SRR13267216	ST398	Pig
SRR21847659	YUSA145	Homo sapiens
SRR21474707	HG003 (Δ0846 suppressor)	Homo sapiens

## Extended Data

Description: Tabular output generated by the AMR algorithm.. Resource Type: Dataset. DOI: 10.22002/my5jc-2zj26

Description: FAIR-compliant Metadata for Assemblies of Six S. aureus genomes.. Resource Type: Text. DOI: 10.22002/f3csp-ahq16
